# A Practical Treatise on the Diseases of the Hair and Scalp

**Published:** 1887-09

**Authors:** 


**A Practical Treatise on the Diseases of the Hair and Scalp.** By GEORGE THOMAS JACKSON, M. D., Instructor in Dermatology, New York Polyclinic. New York: E. B. Treat, 771 Broadway. 1887. Price, $2.75.

In this book of some 400 pages, the physician will find a good summary of the present knowledge concerning the diseases of the hair and scalp. As the author has given special attention to the diagnosis and treatment of these affections, the purchaser will find the book a very valuable and convenient one. The knowledge and information easily had from it on any subject, within the scope of the work, could not otherwise be obtained without consulting many books and expenditure of much time and labor.

